# Measurement of Calprotectin (S100A8/A9) in the Saliva of Pigs: Validation Data of A Commercially Available Automated Assay and Changes in Sepsis, Inflammation, and Stress

**DOI:** 10.3390/ani13071190

**Published:** 2023-03-29

**Authors:** María José López-Martínez, Silvia Martínez-Subiela, José Joaquín Cerón, Alba Ortín-Bustillo, Guillermo Ramis, Marina López-Arjona, Silvia Martínez-Miró, Edgar García Manzanilla, Peter David Eckersall, Fernando Tecles, Damián Escribano, Alberto Muñoz-Prieto

**Affiliations:** 1 Interdisciplinary Laboratory of Clinical Analysis (INTERLAB-UMU), Department of Animal Medicine and Surgery, Veterinary School, Regional Campus of International Excellence Mare Nostrum, University of Murcia, 30100 Murcia, Spain; mariajose.lopez28@um.es (M.J.L.-M.); silviams@um.es (S.M.-S.); jjceron@um.es (J.J.C.); alba.ortinb@um.es (A.O.-B.); david.eckersall@glasgow.ac.uk (P.D.E.); ftecles@um.es (F.T.); alberto.munoz@um.es (A.M.-P.); 2 Department of Animal Production, Veterinary School, Regional Campus of International Excellence Campus Mare Nostrum, University of Murcia, 30100 Murcia, Spain; guirramis@um.es (G.R.); silviamm@um.es (S.M.-M.); 3 Department of Animal and Food Science, Universitat Autònoma de Barcelona, 08193 Bellaterra, Spain; marina.lopez.arjona@uab.cat; 4 Pig Development Department, The Irish Food and Agriculture Authority, Teagasc, Moorepark, P61 C996 Fermoy, Ireland; egmanzanilla@gmail.com; 5 School of Veterinary Medicine, University College Dublin, Belfield, D04 W6F6 Dublin, Ireland; 6 School of Biodiversity, One Health and Veterinary Medicine, University of Glasgow, Bearsden Rd., Glasgow G61 1QH, UK

**Keywords:** S100A8/A9, calprotectin, automated assay, pig, saliva, stress, sepsis

## Abstract

**Simple Summary:**

Calprotectin (CALP, S100A8/A9) is a calcium and zinc-binding protein involved in inflammation that has a wide range of proinflammatory functions, such as cytokine production and regulation of leukocyte adhesion, migration, and phagocytosis. The objective of this study was to validate a commercially available assay for the measurement of CALP in the saliva of pigs and study the variations of this analyte due to sepsis, non-septic inflammation, and stress. The assay showed adequate precision and accuracy for the measurements of CALP in the saliva of pigs. In addition, this protein showed significant increases in the saliva of pigs with sepsis as well as after a stressful situation in our experimental conditions, being the increase in the stress of lower magnitude than in sepsis. Based on these results, CALP can be measured in the saliva of pigs and could be a potential biomarker of health and welfare in this species.

**Abstract:**

Calprotectin (CALP, S100A8/A9), also named myeloid-related protein 8/14, is a dimer complex of S100A8 and S100A9 that belongs to the S-100 protein family. It is involved in inflammation and has a wide range of proinflammatory functions, such as cytokine production and regulation of leukocyte adhesion, migration, and phagocytosis. In humans, CALP traditionally can be measured in faeces, serum, and saliva as a biomarker of inflammation and sepsis. The objective of this study was to validate an automated assay for CALP measurements in the saliva of pigs, having the advantage of the use of a non-invasive sample that is easy to collect. The assay was precise and accurate. CALP in saliva measured by this assay showed significant changes depending on the hour of the day. It also showed significant increases in the saliva of pigs after the administration of lipopolysaccharide (LPS), and showed a rise, although with increases of lower magnitude, after a stressful stimulus. Further studies should be made to gain knowledge about the possible practical applications of the measurements of CALP in the saliva of pigs as a biomarker to evaluate the animals’ health and welfare.

## 1. Introduction

Calprotectin (CALP, S100A8/A9), also named myeloid-related protein 8/14, is a dimer complex of S100A8 and S100A9 which belongs to the S-100 protein family [1]. It is a calcium and zinc-binding protein involved in inflammation and has a wide range of proinflammatory functions, such as cytokine production and regulation of leukocyte adhesion, migration, and phagocytosis [2].

Currently, there is an increasing interest in the use of saliva as a biological sample since it has the advantage of being a non-invasive sample that is easy to collect. This is highly relevant in pigs, a species in which blood sampling is difficult, highly stressful, and painful for the animal. The collection of saliva can be easily made by farm personnel, allowing a more frequent analysis and monitoring to take place at the farm level. Currently, one of the main uses of saliva is for the diagnosis and detection of infectious diseases, but saliva can also be used to measure biomarkers that can provide information on stress, inflammation, immune response, and redox homeostasis [3]. Therefore, saliva contains a source of analytes with the potential to assess the effect of different husbandry conditions and also to evaluate the homeostasis in swine.

In humans, CALP traditionally has been measured in faeces for the detection of inflammatory bowel disease and in serum as a biomarker of inflammation and sepsis [4]. However, it can also be quantified in saliva, where CALP has been reported to be increased in patients with active inflammatory bowel disease, suggesting that intestinal inflammation leads to increases in CALP in this sample type [5]. In pigs, CALP protein levels have been measured in faeces, increasing in animals with colitis [6,7,8]. In addition, variations in gene expression for calprotectin have also been found in the ileum after oral vaccination against *E. coli* [9]. However, to the authors’ knowledge, there are no reports about the measurement of CALP in saliva, despite the advantages that this sample type has.

The objectives of this report were to perform an analytical validation of an automated commercially available assay for the measurement of CALP in the saliva of pigs and to determine if it is influenced by possible variations due to the sampling time during the day. In addition, the variations of this analyte due to sepsis, non-septic inflammation and stress were studied. For this purpose, the CALP concentration was measured in the saliva of pigs with three different conditions: sepsis experimentally induced by inoculation with lipopolysaccharide (LPS), non-septic inflammation induced by turpentine injection and a situation of stress such as staying at the lairage for 4 h after arrival at the slaughterhouse. In the models of LPS and turpentine, the correlation between values of CALP in saliva and CALP and C-reactive protein (CRP) in serum was studied. It is expected that this information will contribute to a better knowledge of the pathophysiology of this protein and its possible use as a biomarker of these conditions in saliva.

## 2. Materials and Methods

### 2.1. Assay

Salivary CALP was determined by the BÜHLMANN fCal Turbo^®^ assay (BÜHLMANN, Laboratories AG, Switzerland) which is an immunoturbidimetric assay using polystyrene nanoparticles coated with polyclonal anti-CALP antibodies. The assay was initially calibrated with serial dilutions of a control material with a known concentration of CALP (Gentian, Moss, Norway) (Calibrator A). Then, a secondary calibrator (Calibrator B) consisting of a pool of saliva samples, in which the CALP concentration was determined with Calibrator A, was used for the complete validation and subsequent sample analysis.

The CALP assay was validated for use in porcine saliva samples using aliquots of the saliva of pigs of the sepsis and non-septic inflammation experiment (Section 2.3). The validation of the assays was performed as follows based on previous procedures [10]:

- Precision: the intra- and inter-assay coefficients of variation (CVs) were assessed using saliva samples with high and low CALP concentrations.
- Accuracy: It was indirectly studied by evaluating linearity after serial dilutions with ultrapure water of saliva and samples with a high level of CALP. Additionally, recovery studies were made to see if there was a matrix effect in the determinations. For this, purified CALP (control material from Gentian, Moss, Norway) was used to spike saliva samples to reach three different CALP concentrations.
- The lower limit of quantification (LLQ): it was calculated as the lowest CALP concentration that the assay was able to determine with an intra-assay CV < 20%.
- Limit of detection (LD): based on the lowest concentration of CALP that the assays can distinguish from a specimen of zero value (ultrapure water), calculated as a mean value plus 3 standard deviations of 12 replicate determinations.

### 2.2. Evaluation of the Sampling in the Daytime

To determine the effect of the time of sampling, surplus saliva samples from 20 male large white pigs aged 5 months from a commercial farm stored from a previous study were used [11]. In these pigs, saliva was sampled at 8 a.m., 12 a.m., 4 p.m., and 8 p.m. within the same day. In this experiment (and in all trials of the report), saliva was collected using Salivette tubes (Salivette, Sarstedt, Aktiengesellschaft & Co., Nümbrecht, Germany). Pigs chewed a sponge for 1 min. Each sponge was then placed in a Salivette tube and stored on ice until arrival at the laboratory. At the laboratory, the tubes were centrifuged at 3000× *g* for 10 min. Saliva samples were kept frozen at −80 °C until analysis.

### 2.3. Experimental Sepsis and Non-Septic Inflammation Induction

To evaluate the changes in CALP concentration caused by sepsis and non-sepsis inflammatory conditions, samples stored from a previous study were used [12]. A total of 15 growing male pigs, in the mid-fattening period, from the University of Murcia Farm, were included in this study. They had water ad libitum and a balanced diet and had a minimum space of 0.65 m^2^ per animal (Council Directive 2001/88/CE of 23 October 2001) with a mean temperature of 24 ± 2 °C. The pigs were 14 weeks old and their median weight was 51.5 kg (interquartile range 48–53 kg).

The pigs were adapted to the experimental conditions for a week and then randomized and divided into three groups of 5 animals each. A control group (n = 5) received saline treatment (2 mL) by an intramuscular route. An LPS group (n = 5) received a single dose of 30 µg/kg LPS from Escherichia Coli (LPS; O55:B5, Sigma-Aldrich, St. Louis, MO, USA) in sterile saline solution by intramuscular injection. A turpentine (TURP) group (n = 5) received 8 mL of TURP (oil of turpentine purified, Sigma–Aldrich) by two 4 mL subcutaneous injections in each front flank per animal. The injections were completed between 8 and 9 a.m.

Saliva and blood samples were obtained 24 h before (baseline) the saline, LPS, or TURP injections and at 6, 24, and 48 h after the treatments. Basal, 24 h, and 48 h samples were obtained at 8 a.m. Saliva was collected and processed as indicated in Section 2.2. Blood samples were collected by venepuncture in an EDTA and plain tubes and serum were separated and stored at −80 °C.

### 2.4. Stress Situation

A total of 13 male pigs at the end-fattening period (5–6 months of age and mean body weight 105.2 ± 7.3 kg) were included in this study. They were from a farm in Southern Spain, where animals were housed in groups of 14 animals per pen (a minimum space of 0.65 m^2^ per animal) and given ad libitum access to a balanced diet and water. The pigs were transported to a commercial slaughterhouse at 15 km from the commercial farm. Transportation was undertaken during the spring of 2022, between 9 and 10 a.m., under commercially accepted conditions. The animals were unloaded on arrival at the slaughterhouse and placed in a lairage area (10 animals per pen) with free access to water. Saliva samples were collected at arrival at the slaughter on the day of transport (T0) (approximately at 10 a.m.) and 4 h after the transport (approximately at 2 p.m.) (T4). The transport of animals was according to the recommendations described in Directive 2001/88/EC, 2001 and Directive 2001/93/EC, 2001.

Saliva samples were obtained as described in Section 2.2. Salivary cortisol concentrations of each animal were measured in these samples with a validated assay [13].

All the experimental procedures of this study were approved by the Ethical Committee on Animal Experimentation (CEEA) of the University of Murcia (A13220196; approval date: 4 March 2021) according to the European Council Directives considering the protection of animals used for experimental purposes. In addition, this study complies with Animal Research: Reporting of In Vivo Experiments (ARRIVE) guidelines for the care and use of animals.

### 2.5. Statistical Analysis

GraphPad Prism software Inc. (GraphPad Prism, version 8 for Windows, Graph Pad Software Inc., San Diego, CA, USA) was used to analyse the data. The D’Agostino and Pearson test was used to evaluate the data distribution giving a nonparametric distribution in all analyses. To analyse the effect of the day on salivary CALP concentrations and the effect of treatment in the LPS, TURP and saline (control) group, the Friedman test was performed, followed by Dunn’s multiple comparison tests to compare the groups over time. The comparison between the groups of pigs after transportation was performed by using the Wilcoxon matched pairs signed rank test. Correlations between salivary CALP and serum CALP and CRP (data of serum CRP in these animals have been reported in a previous study [12]) were analysed through the non-parametric Spearman correlation test. Moreover, in the stress situation, the correlation between salivary CALP and cortisol was evaluated by the same test. A correlation was strong when the correlation coefficient was ≥0.7. The results were considered significant if *p*-values were <0.05.

## 3. Results

### 3.1. Saliva Calprotectin Assay Validations

The evaluation of precision showed mean intra- and inter-assay CVs of 3.50 and 4.79% for salivary CALP (Table 1). In the accuracy assessment, we observed recovery rates ranging from 110% to 116.7% for salivary CALP measurements (Table 2). Furthermore, the serial dilution of saliva samples with a high concentration of CALP showed linear regression equations with a coefficient of correlation close to 1 (Supplementary Material: Figure S1). The LLQ was set at 0.01 mg/L for salivary CALP, and the LD of the assay could not be calculated since all measurements with ultrapure water gave a value of zero.

### 3.2. Evaluation of the Sampling Time on the Day

**Figure 1:**
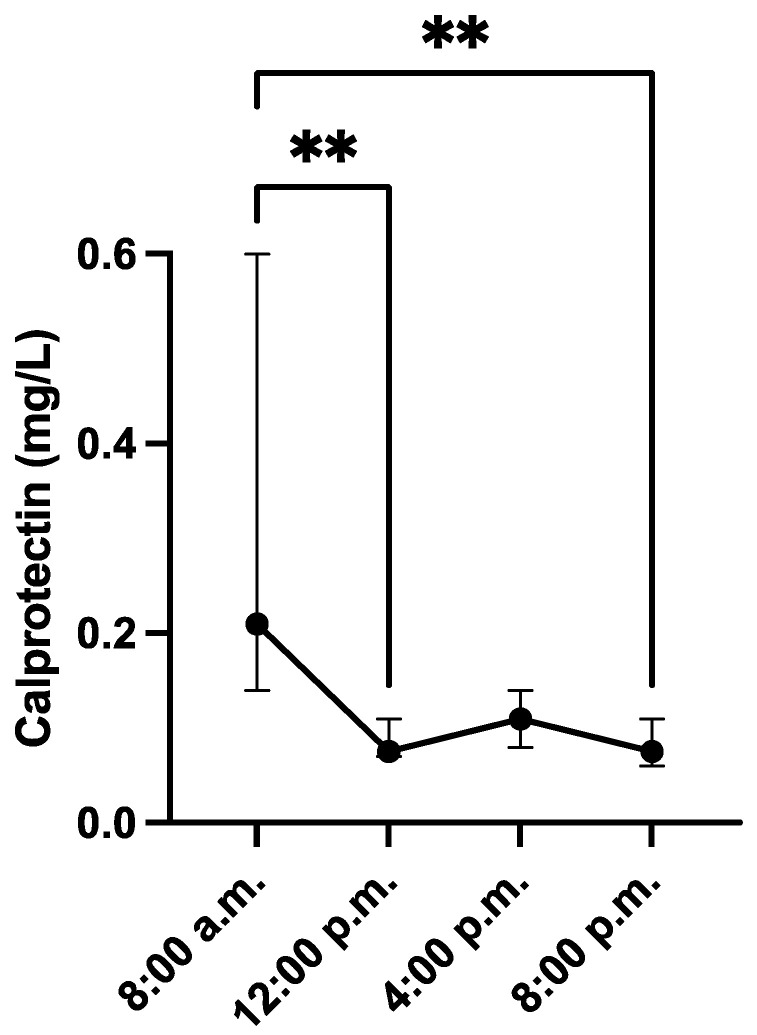
Results of salivary calprotectin concentrations in 20 male pigs obtained at different hours of the day. ** *p*-value < 0.01. Dots are representing median values and whiskers the 95% CI.

The CALP concentrations in saliva showed a tendency to decrease during the day, being these changes significantly at 12 p.m. (median = 0.07 mg/L, range = 0.03–0.30 mg/L) and 8 p.m. (median = 0.07 mg/L, range = 0.03–0.72 mg/L) if compared with values at 8 a.m. (median = 0.21 mg/L, range = 0.01–0.96 mg/L) (Figure 1).

### 3.3. Experimental Sepsis and Non-Septic Inflammation Induction

**Figure 2:**
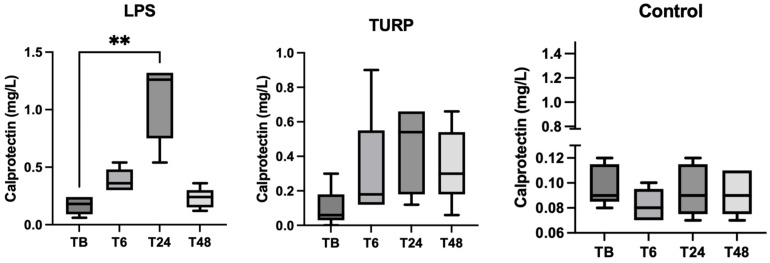
Changes in the salivary calprotectin concentrations of pigs after lipopolysaccharide (LPS), turpentine (TURP) or saline (control) injection. The plots show medians (line within box), 25th and 75th percentiles (boxes) and min and max values (whiskers). Asterisks indicate a statistically significant difference: ** *p* < 0.01.

In saliva, the CALP levels were significantly higher in pigs at 24 h after LPS injection (median = 1.26 mg/L, range = 0.54–1.32 mg/L) compared to basal (pre-treatment) levels (median = 0.18, range = 0.06–0.24 mg/L) (*p* = 0.005), being reduced to nearly basal levels at 48 h (Figure 2). In the case of the administration of TURP, a tendency of increase was observed after the inoculation, but the changes were not significant. The effect of saline injection did not cause any variation in salivary calprotectin levels.

**Figure 3:**
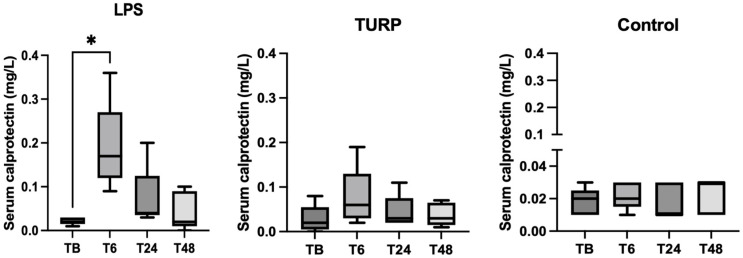
Changes in the serum calprotectin concentrations of pigs after lipopolysaccharide (LPS), turpentine (TURP) or saline (control) injection. The plots show medians (line within box), 25th and 75th percentiles (boxes) and min and max values (whiskers). * *p*-value < 0.05.

In serum, pigs subjected to LPS injection showed a significant increase in CALP concentrations at 6 h (median = 0.17 mg/L, range = 0.09–0.36 mg/L) compared with basal values (median = 0.02 mg/L, range = 0.01–0.03 mg/L) (*p* = 0.01). In the TURP group, there were no significant changes in the pairwise comparison although a tendency of increase at 6 h and 24 h compared with the control group was observed (Figure 3).

The correlation study showed that salivary and serum CALP concentrations were positively correlated (r = 0.283, *p* = 0.02). In addition, serum CRP concentration values were significantly positively correlated with both salivary (r = 0.665, *p* = 0.01) and serum CALP concentrations (r = 0.418, *p* < 0.001). All these correlations were below 0.7 which is considered the threshold for a strong correlation.

### 3.4. Stress Situation

**Figure 4:**
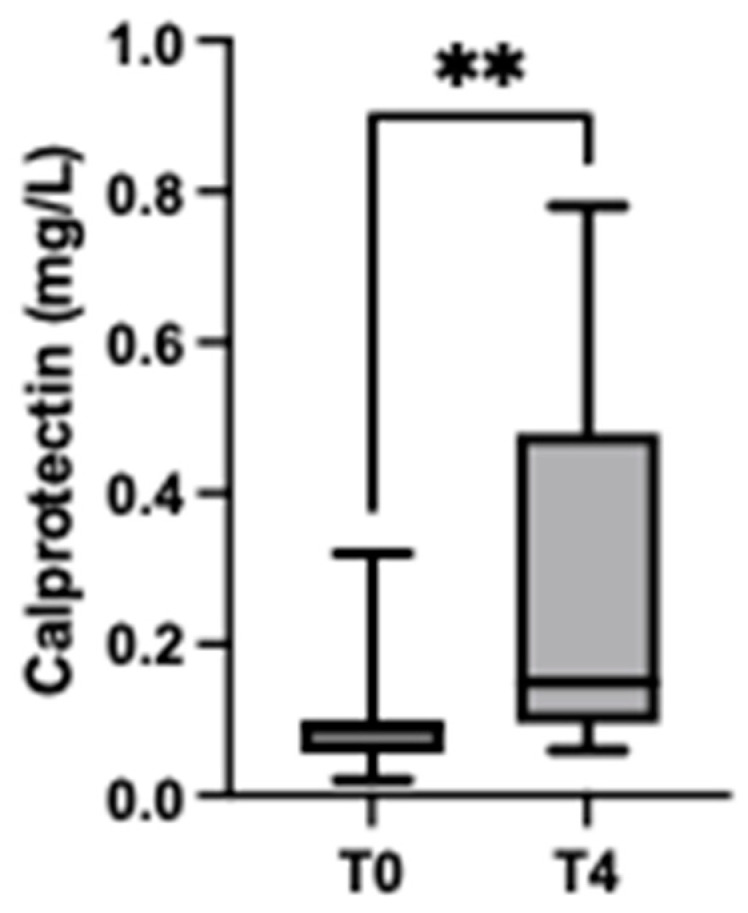
Concentrations of calprotectin in pigs at the arrival to the slaughterhouse (T0) and 4 h after transportation (T4). The plots show medians (line within box), 25th and 75th percentiles (boxes) and min and max values (whiskers). ** *p*-value < 0.01.

The concentrations of CALP were significantly higher in pigs 4 h after arrival at the slaughterhouse (median = 0.15 mg/L, range = 0.06–0.78 mg/L) compared with basal values (median = 0.09 mg/L, range = 0.02–0.32 mg/L) (*p* = 0.002) (Figure 4).

Values of salivary CALP showed a significant positive but weak correlation with salivary cortisol concentrations in the pigs included in this comparison (r = 0.396, *p* = 0.04)

## 4. Discussion

In this report, the validation of an immunoturbidimetric assay for CALP in porcine saliva which allows its automated quantification was performed. The assay was found to be valid for use in non-invasively collected saliva from pigs. There are various commercially available kits for the detection of CALP in faeces and serum, from ELISAs to point-of-care assays. This assay was selected for validation because it has also been previously described to measure CALP in pig faeces, and therefore it has already been shown to be able to detect CALP in this species. Furthermore, it is an automated assay suitable for veterinary clinical pathology laboratories [6]. This assay uses polystyrene nanoparticles coated with polyclonal antibodies, which form complexes with CALP that can be detected by light absorbance using automated clinical chemistry analysers, therefore it is a rapid assay allowing high sample throughput.

Two modifications were made in this assay. One was the use of a solution of purified human CALP as Calibrator A to initially measure in saliva samples of pigs. With this calibrator, the units for the concentration of CALP in saliva were assessed in mg/L instead of µg/g as is the concentration of the original calibrator of the kit. The second modification was the calibration of this assay with Calibrator B, which consists in a pooled saliva sample with a known concentration of CALP, in order to reduce the possible matrix effect. Overall, the modified assay of our study was precise and linear in saliva, being in line with the previous report in which this assay could detect CALP in the faeces of pigs [6]. 

The increases after LPS administration found in our study indicate that CALP increases in sepsis, which is in line with the findings in humans where increases in this protein in serum have been found in patients with sepsis. This allows early diagnosis of sepsis on intensive care unit (ICU) admissions in adults [4] as well as infants [14], and is considered to be a tool that can aid timely sepsis management reducing mortality rates and avoiding unnecessary antibiotic treatment, thus improving antibiotic stewardship. The increases found in sepsis in pigs were higher than in the non-sepsis inflammatory condition. This would agree with previous reports in humans which found higher values in sepsis compared with patients with non-septic inflammation [15]. Further studies with a larger number of individuals would be recommended to evaluate the ability of CALP to differentiate between these two processes.

In our study, significant CALP increases in both pigs’ saliva and serum after LPS administration were detected. The values of CALP in serum were lower than in saliva in agreement with previous reports in humans [5,16], so a possible production of this protein in saliva could be hypothesised, as it has been described in mice [15]. This could also be a reason for the weak correlation between the concentrations of saliva and serum of CALP in our experimental conditions. Peak increases in serum were earlier than in saliva, and further studies should be undertaken to elucidate the mechanism involved, and also to evaluate which sample type could be more sensitive to detect septic conditions. In general, the values of CALP in serum were small with the absorbances of the assay at the low end of the range; therefore, in the future, the use of other CALP assays could be explored that could generate higher assay signals with this sample type.

An increase in the values of CALP was found after a situation of stress consisting of a 4-h stay at the slaughterhouse. This stress is due to new situations that face the pigs at the slaughterhouse, with various stressful stimuli such as strange sounds and mixing with unfamiliar pigs [17]. Previous reports have indicated that stress can increase faecal CALP in humans, suggesting that this increase could be related to the regulation of the immune system by stress [18], while other reports have associated these increases with the activation of inflammatory processes in the gut that occurs in stressful conditions [19,20]. The increases found in our study in the stress due to pre-slaughter lairage were lower than those found in sepsis, however further studies with other types of stress should be performed to evaluate if in some cases the increases could be high enough to mask a septic situation.

When the assay was applied for the measurement of saliva CALP at different times of the day, the highest values were found at 8 a.m. Changes in the values of some analytes in the saliva of pigs depending on the hour of the day in which the sample is collected have been previously described [11]. In the experiment of septic and non-septic inflammation induction of this report, samples were obtained at 8 a.m. for the pre-treatment basal as well as the 24 h and 48 h post-treatment samples, whereas the sample collected at 6 h was obtained at 2 p.m. In this case, the magnitude of the change in healthy animals between 8 a.m. and 2 p.m. was not so pronounced as the increases found in the LPS-injected pigs. In the trial of stress induction, it could be postulated that a decrease in calprotectin concentrations would occur between 10 a.m. and 2 p.m. in animals without stress and therefore reinforces the increase found in our study at 2 p.m. after the stressful condition. In any case, sample collection time should be considered an important factor during practical processing, and it would be recommended for the analysis of calprotectin in saliva, if possible, to take the samples at the same time of day. In addition, the study of the possible variations of CALP in saliva during different times of the day in animals with sepsis or different diseases and stress conditions should be assessed to identify if the pattern found here for healthy pigs is also followed in pigs with such conditions.

This study has some limitations. It is important to point out that the results here have been obtained with a specific assay and other assays could provide different values for CALP, as have been reported in humans using different assays such as enzyme-linked immunoassays, automated fluoroimmunoassays, immunochromatographic tests, chemiluminescent or immunoturbidimetric assays [21,22,23]. Overall, each assay should be independently validated from an analytical and clinical point of view before use in each species under investigation. Additional studies should be made to elucidate the influence in saliva CALP of possible sources of variation such as breed, age, season, or productive condition. Moreover, the trials of sepsis and non-septic inflammation induction were performed with a limited number of animals and should be confirmed in a larger number of individuals. Furthermore, the evaluation of the potential application of CALP in saliva as a biomarker in different diseases within a large population of animals should be performed. In addition, it should be determined if using porcine CALP as a standard could increase its diagnostic value in pigs, as has been reported with other assays, such as the CRP assay in serum when also using kits designed to measure it in humans [24].

## 5. Conclusions

It can be concluded that CALP can be measured in the saliva of pigs with the assay evaluated in this study and that its concentration showed variations depending on the time of the day in which the sample was obtained. In addition, it was found that CALP increases in the saliva of pigs with sepsis showing, in our experimental conditions, increases of higher magnitude than in pigs with non-septic inflammation. Finally, this protein increases after a stressful situation consisting of a stay of 4 h in lairage at the slaughterhouse, although these increases were of lower magnitude than those in sepsis. Further studies should be made to gain knowledge about the possible practical applications of the measurements of CALP (S100 A8/A9) in the saliva of pigs as a biomarker to evaluate the animals’ health and welfare.

## Figures and Tables

**Table 1:** Precision study of the salivary calprotectin assays. (SD, standard deviation; CV, coefficient of variation).

Method	Comparison	Samples	Mean (mg/L)	SD (mg/L)	CV (%)
Saliva	Intra-assay	High	1.54	0.03	2.14
		Low	0.55	0.02	4.86
	Inter-assay	High	1.67	0.04	4.26
		Low	0.43	0.03	6.23

**Table 2:** Recovery study of the calprotectin assay in saliva.

% Analyte		Expected (mg/L)	Observed (mg/L)	Recovery (%)
100	0	0.72	0.72	100
75	25	0.66	0.60	110
50	50	0.54	0.48	112.5
25	75	0.42	0.36	116.7
0	100	0.24	0.24	100

## Data Availability

The data presented in this study are available on request from the corresponding author.

## Supplementary Materials

The following supporting information can be downloaded at: https://www.mdpi.com/article/10.3390/ani13071190/s1, Supplementary Figure S1. Linearity under the dilution of saliva calprotectin assay.

## References

[1] Manolakis A.C., Kapsoritakis A.N., Tiaka E.K., Potamianos S.P. (2011). Calprotectin, Calgranulin C, and Other Members of the S100 Protein Family in Inflammatory Bowel Disease. Dig. Dis. Sci..

[2] Yang Y., Shen L., Xu M., Chen L., Lu W., Wang W. (2021). Serum Calprotectin as a Prognostic Predictor in Severe Traumatic Brain Injury. Clin. Chim. Acta.

[3] Cerón J.J., Contreras-Aguilar M.D., Escribano D., Martínez-Miró S., López-Martínez M.J., Ortín-Bustillo A., Franco-Martínez L., Rubio C.P., Muñoz-Prieto A., Tvarijonaviciute A. (2022). Basics for the Potential Use of Saliva to Evaluate Stress, Inflammation, Immune System, and Redox Homeostasis in Pigs. BMC Vet. Res..

[4] Gao R.-Y., Jia H.-M., Han Y.-Z., Qian B.-S., You P., Zhang X.-K., Li W.-X., Huang L.-F. (2022). Calprotectin as a Diagnostic Marker for Sepsis: A Meta-Analysis. Front. Cell Infect. Microbiol..

[5] Majster M., Almer S., Boström E.A. (2019). Salivary Calprotectin Is Elevated in Patients with Active Inflammatory Bowel Disease. Arch. Oral Biol..

[6] Barbosa J.A., Rodrigues L.A., Columbus D.A., Aguirre J.C.P., Harding J.C.S., Cantarelli V.S., Costa M.D.O. (2021). Experimental Infectious Challenge in Pigs Leads to Elevated Fecal Calprotectin Levels Following Colitis, but Not Enteritis. Porc. Health Manag..

[7] Bogere P., Choi Y.J., Heo J. (2019). Optimization of Fecal Calprotectin Assay for Pig Samples. J. Agric. Life Sci..

[8] Sánchez-Uribe P., Romera-Recio E., Cabrera-Gómez C.G., Hernández-Rodríguez E.V., Lamrani Á., González-Guijarro B., de Pascual-Monreal C., Mendonça-Pascoal L., Martínez-Alarcón L., Ramis G. (2022). Effect of β-Mannanase Addition during Whole Pigs Fattening on Production Yields and Intestinal Health. Animals.

[9] Ramis G., Pérez-Esteruelas L., Gómez-Cabrera C.G., de Pascual-Monreal C., Gonzalez-Guijarro B., Párraga-Ros E., Sánchez-Uribe P., Claver-Mateos M., Mendonça-Pascoal L., Martínez-Alarcón L. (2022). Oral and Parenteral Vaccination against Escherichia Coli in Piglets Results in Different Responses. Animals.

[10] Christensen M., Jacobsen S., Ichiyanagi T., Kjelgaard-Hansen M. (2012). Evaluation of an Automated Assay Based on Monoclonal Anti-Human Serum Amyloid A (SAA) Antibodies for Measurement of Canine, Feline, and Equine SAA. Vet. J..

[11] Ortín-Bustillo A., Contreras-Aguilar M.D., Rubio C.P., Botia M., Cerón J.J., López-Arjona M., Martínez-Subiela S., Escribano D., Tecles F. (2022). Evaluation of the Effect of Sampling Time on Biomarkers of Stress, Immune System, Redox Status and Other Biochemistry Analytes in Saliva of Finishing Pigs. Animals.

[12] López-Martínez M.J., Escribano D., Ortín-Bustillo A., Franco-Martínez L., González-Arostegui L.G., Cerón J.J., Rubio C.P. (2022). Changes in Biomarkers of Redox Status in Saliva of Pigs after an Experimental Sepsis Induction. Antioxidants.

[13] Escribano D., Fuentes-Rubio M., Cerón J.J. (2012). Validation of an Automated Chemiluminescent Immunoassay for Salivary Cortisol Measurements in Pigs. J. Vet. Diagn. Investig..

[14] Pirr S., Dauter L., Vogl T., Ulas T., Bohnhorst B., Roth J., Viemann D. (2021). S100A8/A9 Is the First Predictive Marker for Neonatal Sepsis. Clin. Transl. Med..

[15] Simm M., Söderberg E., Larsson A., Castegren M., Nilsen T., Eriksson M., Lipcsey M. (2016). Performance of Plasma Calprotectin as a Biomarker of Early Sepsis: A Pilot Study. Biomark. Med..

[16] Kamp K., Clark-Snustad K., Saad K., Tolentino E., Heitkemper M., Dey N., Gui S., Lee S.D. (2022). Correlation of Fecal, Plasma, Serum, and Salivary Calprotectin to Endoscopic and Histologic Outcomes in Patients with Crohn’s Disease. Gastroenterology.

[17] López-Arjona M., Escribano D., Mateo S.V., Contreras-Aguilar M.D., Rubio C.P., Tecles F., Cerón J.J., Martínez-Subiela S. (2020). Changes in Oxytocin Concentrations in Saliva of Pigs after a Transport and during Lairage at Slaughterhouse. Res. Vet. Sci..

[18] Xu W., Zhang Y., Zhao W., Chen J., Maas K., Hussain N., Henderson W.A., Cong X. (2022). Trends of Fecal Calprotectin Levels and Associations with Early Life Experience in Preterm Infants. Interdiscip. Nurs. Res..

[19] González-Moret R., Cebolla A., Cortés X., Baños R.M., Navarrete J., de la Rubia J.E., Lisón J.F., Soria J.M. (2020). The Effect of a Mindfulness-Based Therapy on Different Biomarkers among Patients with Inflammatory Bowel Disease: A Randomised Controlled Trial. Sci. Rep..

[20] Sauk J.S., Ryu H.J., Labus J.S., Khandadash A., Ahdoot A.I., Lagishetty V., Katzka W., Wang H., Naliboff B., Jacobs J.P. (2023). High Perceived Stress Is Associated with Increased Risk of Ulcerative Colitis Clinical Flares. Clin. Gastroenterol. Hepatol..

[21] Oyaert M., Boel A., Jacobs J., Van den Bremt S., De Sloovere M., Vanpoucke H., Van Hoovels L. (2017). Analytical Performance and Diagnostic Accuracy of Six Different Faecal Calprotectin Assays in Inflammatory Bowel Disease. Clin. Chem. Lab. Med..

[22] Juricic G., Brencic T., Tesija Kuna A., Njegovan M., Honovic L. (2019). Faecal Calprotectin Determination: Impact of Preanalytical Sample Treatment and Stool Consistency on within- and between-Method Variability. Biochem. Med..

[23] Labaere D., Smismans A., Van Olmen A., Christiaens P., D’Haens G., Moons V., Cuyle P.-J., Frans J., Bossuyt P. (2014). Comparison of Six Different Calprotectin Assays for the Assessment of Inflammatory Bowel Disease. United Eur. Gastroenterol. J..

[24] Muñoz-Prieto A., Tvarijonaviciute A., Escribano D., Martínez-Subiela S., Cerón J.J. (2017). Use of Heterologous Immunoassays for Quantification of Serum Proteins: The Case of Canine C-Reactive Protein. PLoS ONE.

