# Supplementary figures and images for: Measurement of Calprotectin (S100A8/A9) in the Saliva of Pigs: Validation Data of A Commercially Available Automated Assay and Changes in Sepsis, Inflammation, and Stress
Source: Animals (Basel). 2023 Mar 29;13(7):1190. doi: 10.3390/ani13071190 (PMC10092961; doi:10.3390/ani13071190)

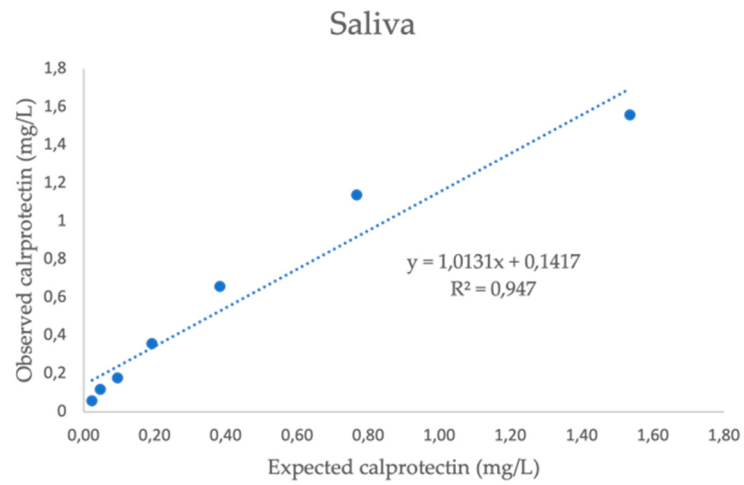

**Supplementary Figure S1.** Linearity under the dilution of saliva calprotectin assay.

Supplement: Supplementary file 1 [file animals-13-01190-s001.zip › animals-2304189-supplementary.pdf]
